# Cytomegalovirus in pregnancy and the neonate

**DOI:** 10.12688/f1000research.10276.1

**Published:** 2017-02-14

**Authors:** Vincent C. Emery, Tiziana Lazzarotto

**Affiliations:** 1 Department of Microbial and Cellular Sciences, School of Biosciences and Medicine, University of Surrey, Guildford, Surrey, UK; 2 Department of Specialized, Experimental and Diagnostic Medicine, University of Bologna, Bologna, Italy

**Keywords:** cytomegalovirus, pathogenesis, pregnancy, antiviral therapy

## Abstract

Congenital cytomegalovirus (CMV) remains a leading cause of disability in children. Understanding the pathogenesis of infection from the mother via the placenta to the neonate is crucial if we are to produce new interventions and provide supportive mechanisms to improve the outcome of congenitally infected children. In recent years, some major goals have been achieved, including the diagnosis of primary maternal CMV infection in pregnant women by using the anti-CMV IgG avidity test and the diagnosis and prognosis of foetal CMV infection by using polymerase chain reaction real-time tests to detect and quantify the virus in amniotic fluid. This review summarises recent advances in our understanding and highlights where challenges remain, especially in vaccine development and anti-viral therapy of the pregnant woman and the neonate. Currently, no therapeutic options during pregnancy are available except those undergoing clinical trials, whereas valganciclovir treatment is recommended for congenitally infected neonates with moderately to severely symptomatic disease.

## Introduction

Cytomegalovirus (CMV) remains a major cause of congenital infection and disease during pregnancy around the world ^1^. Our understanding of the pathogenesis of CMV during pregnancy continues to improve through the application of new technologies and interventional studies. However, there remain a number of outstanding questions relating to CMV infection in pregnancy, especially in the context of women who are already seropositive for CMV where multiple strains may be present in the face of a strong T- and B-cell immune response, the comparative pathogenesis in developed and developing countries, and the optimal therapeutic strategies to be deployed. Recently, there has been significant interest in establishing associations between genetic variants and strain pathogenicity of CMV. In 2013, Renzette *et al*. created the most detailed map of human CMV *in vivo* evolution to date and demonstrated that viral populations can be stable or rapidly differentiate, depending on the host environment ^2^. Furthermore, in 2015, Sijmons *et al*. provided an important compendium of data concerning human CMV strain diversity ^3^. These studies support the hypothesis that human CMV strains may vary in virulence, tropism, and pathogenic potential, which in turn is probably related to the genetic variability exhibited in key genes important for pathogenesis among wild-type CMV strains. Identification of specific, more highly pathogenic, CMV variants could provide clinically useful information ^3^.

The present article will survey the progress that has been made in these areas, particularly over the last 4 years, and provide a succinct update on how our understanding of CMV in pregnancy has matured in recent years and the potentially beneficial effects of anti-viral therapy in managing congenital CMV infection and disease.

## Cytomegalovirus in pregnancy: risk factors and epidemiology

**Figure 1.**
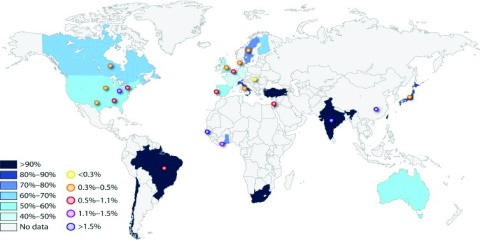
Global cytomegalovirus (CMV) seroprevalence levels and incidence of congenital CMV infection. Worldwide CMV seroprevalence rates among women of reproductive age and birth prevalence of congenital CMV infection (reproduced from 1). CMV seroprevalence rates are shown in different shades of colour, and congenital CMV birth prevalence rates are shown by the circles.

CMV seroprevalence across the globe varies substantially both between and within countries ^4– 7^ ( Figure 1). As a rule of thumb, lower socioeconomic groups have a higher incidence of CMV exposure and resource-poor countries also have higher seroprevalence levels with infection (from 84% to 100% IgG-positive) frequently acquired early in life ^8^. These epidemiological patterns have a direct impact on the incidence of congenital infection and disease. For example, the classic high-risk setting for congenital CMV infection and particularly disease is where a seronegative mother becomes infected during pregnancy (in particular, during the first trimester) and transmits the virus to the foetus. In this situation, transmission to the foetus occurs in 30–35% of cases and congenital disease in around 10–15% of those born with congenital infection ^6^. In contrast, in women who are already seropositive, reactivation or reinfection gives rise to a foetal infection rate of about 1.2%, which whilst much lower than primary infection acts to be the main contributor to the total number of congenital infections (and disease) worldwide ^9– 11^. Lastly, increasing observations demonstrate the risk for symptomatic infection at birth, and sequelae, especially hearing loss, are similar upon primary and non-primary maternal CMV infection ^8, 12– 15^.

In terms of pathogenesis, in children with symptomatic disease, approximately 4% will die *in utero* or shortly after birth and this is usually because of significant neurological damage and multi-organ failure ^16^. Of the remainder, about 60% will have cognitive defects; sensorineural hearing loss (SNHL) and neurological impairment are two common manifestations ^16^. Indeed, SNHL, which is prevalent at about 35%, is a progressive disease, and, even in neonates born with asymptomatic infection, there is now increasing evidence that a significant subset of these will develop SNHL ^17– 19^. Thus, early identification of children at risk of progressive SNHL is a priority, as interventions may provide a substantial benefit (an aspect that will be discussed later).

In CMV seropositive women, the risk factors for congenital infection are less well defined, although in a recent study of pregnant Polish women, around 22% of seropositive women who transmitted to their neonate were infected with multiple CMV strains as judged by glycoprotein B genotyping ^20^. Additional studies by targeted sequencing demonstrated that other genes of CMV have highly variable regions including proteins gN, gO, gH, and UL144 ^2, 3^. However, the role that mixed genotype infection plays in transmission and the immune response against these different strains has not been fully investigated.

In healthy humans, the T-cell immune response against CMV is both multi-specific and high frequency in both CD4 and CD8 compartments ^21^. In many studies of transplant recipients, a high frequency of functional (that is, interferon gamma [IFN-γ]-producing) CMV-specific CD8 together with CD4 helper cells has been shown to be protective against high-level CMV replication and also disease ^22– 25^. Thus, the assumption might be that in pregnant women a similar T-cell phenotype may be protective against CMV disease. Recent data question this hypothesis; with IFN-γ ELISpot assay and CMV IgG avidity testing, it was shown that women who transmitted to their offspring were more likely to have a higher T-cell response and that the combination of low CMV IgG avidity and high ELISpot values gave an area under the curve of 0.87 ^26^.

Eldar-Yedidia *et al*. proposed a novel normalisation method testing the individual IFN-γ response to CMV (IFN-γ relative response, or RR) detected by the QuantiFERON assay and found that the group of women with a low CMV IFN-γ RR did not transmit the virus to the foetus ^27^. However, Forner *et al*. showed that the results of CMV cell-mediated immunity obtained with the ELISpot assay were more significantly associated with the risk of CMV transmission ******** compared with those obtained with the QuantiFERON test ^28^. Further studies in a larger number of CMV-infected pregnant women should be performed in order to verify the prognostic efficacy of determining the maternal CMV-specific T-cell immune response. Finally, data using a Rhesus animal model of CMV have clearly demonstrated that, in the absence of CD4 T cells, more severe CMV disease was observed in the offspring ^29^.

## Advances in screening for cytomegalovirus in pregnancy

Screening of mothers for CMV IgG on a routine basis has not been universally adopted, although at present eight European countries have adopted a *de facto* screening ^30, 31^. One of the stated reasons why such an approach is not warranted is the incidence of congenital infection and disease amongst children born to women who are already CMV IgG positive. However, if there is clinical suspicion of primary CMV infection, appropriate testing of antenatal samples for seroconversion and investigating IgG avidity levels are standard practice ^32^. To assess the risk of transmission to the foetus, prenatal testing of amniotic fluid at 20–21 weeks of gestation by real-time polymerase chain reaction (PCR) has also been investigated ^33– 40^. The foetal CMV diagnosis is reliable: a negative result in the amniotic fluid can rule out foetal infection with a high degree of certainty. Positive results in amniotic fluid identify CMV-infected foetuses but do not discriminate those infants who will have symptoms at birth. Studies have shown that low viral loads in amniotic fluid are associated with a lower risk of congenital disease, but it is clear that the positive and negative predictive values of qualitative and quantitative PCR on amniotic fluid are not sufficiently robust for routine deployment and should be considered with caution ^41, 42^.

## Advances in understanding the role of the placenta in infection

In recent years, our understanding of the complex interaction between the placenta and CMV and how it relates to congenital infection has improved significantly. Of particular note here is the work of the Pereira group. The recent observations show that CMV particularly infects amniotic membranes ^43^, impairs cytotrophoblast-induced lymphangiogenesis and vascular remodelling in the placenta, and arrests the correct development of human trophoblast progenitor cells, thus interfering with the earliest stages in the growth of new villi. This results in increased hypoxia, which ultimately contributes to restriction in foetal growth ^44^. In fact, immunohistochemical and virological studies of placental tissues suggested that severe placental infection was associated with diffuse villitis and necrosis, consistent with functional impairment and possible consequent hypoxic cerebral damage ^45^.

CMV infection also appears to persist in the amniotic epithelial cells and has been associated with increased expression of the anti-apoptotic proteins *survivin* and Bclx-l through both STAT-3-dependent and -independent mechanisms. ^46^. In addition, CMV has been shown to inhibit Wnt5a-stimulated migration of trophoblasts through increasing the expression of the WNT receptor ROR2 ^47^.

CMV infection induces an innate immune response in the placenta, significantly altering the decidual cytokine and chemokine environment. In particular, Hamilton *et al*. highlighted how CMV infection modulates the placental immune environment, suggesting CMV-induced upregulation of monocyte chemoattractant protein-1 (MCP-1) and tumour necrosis factor-alpha (TNF-α) expression as a potential initiator or exacerbator (or both) of placental and foetal injury ^48^. In CMV-infected decidual cultures, there is a predominant induction of INF-γ and inducible protein 1 (IP-1) expression, reflecting the immune activation generated upon CMV infection ^49^.

## Advances in risk stratification of infected neonates

A comprehensive analysis by Cannon *et al*. ^50^ assessed each of the sequelae of congenital CMV and the evidence for different interventions making a positive impact. This study concluded that there was good evidence that non-pharmaceutical interventions for children with delayed hearing loss by 9 months of age would have an impact and that a moderate effect of pharmaceutical interventions would be observed on hearing loss between 9 and 24 months and on CMV-related cognitive deficits. However, the evidence for interventions affecting children with hearing loss of neurological impairment occurring after 24 months was weak. On this basis, the authors proposed that a combination of newborn screening and early detection and interventions would benefit thousands of children with congenital CMV in the USA alone.

At present, no European country routinely screens for congenital CMV infection. In four states in the USA, the efforts of non-profit organisations have determined the introduction and passage of legislation for infant CMV screening. Attention over the last decade has turned to the use of dried blood spots, since these are routinely taken to diagnose biochemical and genetic disorders in the newborn. However, the sensitivity of this approach using PCR-based detection methods is highly variable (28–100%), even when large sample sizes have been used ^51– 53^. Further improvements in sensitivity can be achieved by standardised procedures of viral DNA extraction and nested PCR approaches, with a recent article showing a sensitivity of 81% ^54^. As an alternative, saliva has been suggested as the sample of choice, but this would require a different sample to be taken at birth, and for routine screening this may prove to be too complex and costly to adopt ^55^.

## Impact of co-infections on cytomegalovirus in pregnancy

A major co-infection which impacts directly on CMV in pregnancy is HIV infection. Recent data highlight that HIV-1/CMV co-infected infants have a high risk of mortality, neurological defects, and HIV disease progression ^56, 57^. Rates of congenital CMV infection in HIV-exposed but uninfected infants in Nairobi have been shown to be 6.3% and this increased to 29% if the infant was HIV infected ^58^. To date, no large systematic studies have investigated the transmission frequency in HIV-infected women of CMV to the neonate in the context of both maternal CMV infection during pregnancy versus post-partum infection, which is known to be prominent through CMV in breast milk. However, one study has used data from a valacyclovir interventional study (for herpes simplex virus [HSV] infection) to investigate its effects on infant CMV acquisition ^59^. Valacyclovir at the doses used for HSV-1 control has a relatively weak anti-viral effect on CMV replication. The upshot of this study was that maternal valacyclovir use had no effect on the timing or acquisition of infant CMV or on breast milk viral loads but that it did reduce cervical CMV shedding. Further studies using high-dose valacyclovir or safer anti-CMV drugs would be warranted in this patient group ^60^. It has also been shown that maternal highly active anti-retroviral therapy (HAART) can reduce vertical transmission of CMV but does not reduce breast milk levels and so would be unlikely to impact on post-natal infection ^61^.

## Advances in anti-viral therapy

The most extensive study to date on the use of anti-viral therapy for symptomatic CMV disease was published in 2015 ^62^. The study enrolled 96 neonates who were randomly assigned to either 6 months or 6 weeks of valganciclovir therapy. The primary endpoint (best ear audiological improvement) was similar between the two arms, although total ear hearing was more likely to have improved and be stable at 12 months in the 6-month valganciclovir arm. Interestingly, the 6-month treatment arm was also associated with significant improvements ( *P* <0.004) in neurodevelopmental scores (improvement in the language-composite component and the receptive-communication scale). This study builds on a previous anti-viral study of intravenous ganciclovir ^63^ and, though encouraging, shows that until we have access to anti-CMV drugs with greater potency and improved side-effect profiles, it is unlikely that this area will move forward rapidly. Currently, valganciclovir treatment for 6 months is recommended for congenitally infected neonates with moderate to severe CMV disease and should be started within the first month of life.

At present, there is no evidence for the potential benefit of treatment of asymptomatic infants or asymptomatically infected children with isolated sensorineural hearing loss (≥20 dB in one or both ears) ^62, 64^. One non-randomised, single-blind clinical trial is currently investigating whether early treatment with valganciclovir of infants up to 12 weeks of age with both congenital CMV infection and SNHL can prevent progression of hearing loss (ClinicalTrials.gov identifier NCT02005822). Two other clinical trials are being undertaken in order to provide evidence for treatment options in congenitally CMV-infected newborns (ClinicalTrials.gov identifiers NCT01649869 and NCT02606266).

A preliminary report on an open-label phase II study of *in utero* treatment of congenital CMV with high-dose valacyclovir (8 g/day) has recently been published. The interim analysis indicates that longer-term exposure to valacyclovir (median of 89 days) decreases foetal viral loads significantly and combined with historical controls decreases the proportion of symptomatic neonates from 57% to 18% ^60^. However, the trial design using historical controls is not optimal and so further, more substantial analysis of this ongoing study is required and there is a need for others to replicate the study design in a controlled way.

## Vaccine development and its potential impact

The basic reproductive number (Ro) for CMV for infections occurring in the developed world is around 2.4, meaning that for herd immunity a vaccine update rate (assuming 100% efficacy) would need to be 59–62% to achieve eradication ^65^. Modelling has shown that if vaccination was started in all toddlers and children aged 12, after 4 years a decline in infected babies occurs (owing to girls immunised at age 12 starting to enter child-bearing age) but that a more rapid decline is observed for babies with congenital infection born to seronegative women and also seropositive women who become re-infected during pregnancy ^66^. To date, only a recombinant CMV glycoprotein B (gB) vaccine administered with the adjuvant MF59 has been evaluated in seronegative women; the vaccine showed an approximately 50% reduction in maternal infection in women vaccinated ^67^. This phase 2 study also observed one congenital infection in the vaccine group compared with three in the placebo arm, although the sample size prevented statistically valid analysis of this observation.

Very recently, a study performed in a guinea pig model demonstrated that immunisation with a novel bivalent vaccine based on a non-replicating lymphocytic choriomeningitis virus (LCMV) vector expressing gB and pp65 did not show interference. Moreover, the bivalent vaccine elicited potent humoral and cellular responses and conferred protection, reducing the magnitude of maternal viremia and improving pup outcomes. These results support further testing of LCMV-vector-based human CMV vaccines in clinical trials ^68^.

An alternative approach using hyperimmune globulin to prevent congenital CMV has also been subjected to a randomised controlled trial ^69^. In this study, 124 pregnant women with primary CMV infection at 5–26 weeks of gestation were randomly assigned to hyperimmune globulin or placebo every 4 weeks until 36 weeks of gestation or until CMV was detected in amniotic fluid. The rate of congenital infection was comparable between the two groups (hyperimmune globulin 33%, placebo 44%; *P* = 0.13). Hyperimmune globulin had no effect on maternal CMV DNA levels in the blood or in time to clearance of DNA from the blood and did not impact on CMV DNA levels in the placenta. However, the results of this randomised placebo-controlled trial showed no agreement with those from a non-randomised study published in 2005 by Nigro *et al*., who showed that the administration of CMV-specific hyperimmune globulin to pregnant women with primary infection significantly decreased the rate of mother-to-foetus transmission, from 40% to 16% ( *P* = 0.04) ^70^. Further studies are needed given the biological plausibility of this approach ^70^.

Currently, two randomised, phase 3 studies of the prevention of congenital infection are underway. One, sponsored by Biotest, is being conducted in Europe, and the second, sponsored by the Eunice Kennedy Shriver National Institute of Child Health and Human Development, is ongoing in the USA (ClinicalTrials.gov identifier NCT01376778).

The hope is that these studies will further aid our understanding of the efficacy and safety of hyperimmune globulin administration as a means of preventing congenital CMV infection.

A systematic review published by Hamilton *et al*. highlighted how, despite a number of case series and case control and observational studies, there is a significant lack of robust clinical trial data examining prophylactic interventions for congenital CMV infection ^71^.

## Conclusions and future prospects

The last few years have seen real progress in understanding the basic biology of CMV in the placenta and the role that ongoing viral replication plays in the pathogenesis of CMV disease in the neonate. However, for a variety of reasons, screening of pregnant women for CMV, whilst supported, has not been implemented globally, and routine surveillance of neonates for evidence of CMV infection requires new methodologies or improvement of the current ones, especially with respect to simpler protocols and lower costs. The therapy of the newborn infant with CMV shows promise, but we need safer and preferably more potent drugs to make a large impact; new drugs such as letermovir are on the horizon, as are active vaccine development programmes, making the future of CMV in pregnancy a very active area of basic and translational research.

## Abbreviations

CMV, cytomegalovirus; gB, glycoprotein B; HSV, herpes simplex virus; IFN-γ, interferon-gamma; LCMV, lymphocytic choriomeningitis virus; PCR, polymerase chain reaction; Ro, basic reproductive number; ROR2, receptor tyrosine kinase-like orphan receptor; RR, relative response; SNHL, sensory neural hearing loss; STAT, signal transducer and activator of transcription.
